# Vascular remodeling enhances high-flow muscle oxygen delivery following aerobic exercise training

**DOI:** 10.3389/fphys.2026.1840439

**Published:** 2026-07-01

**Authors:** E. Maufroy, C. Rigaut, C. Maufroy, N. Baeyens, G. Deboeck

**Affiliations:** 1 Research Unit in Rehabilitation Sciences, Faculty of Human Movement Sciences, Université libre de Bruxelles (ULB), Brussels, Belgium; 2 Transferts, Interfaces and Processes, Ecole Polytechnique de Bruxelles, Université libre de Bruxelles (ULB), Brussels, Belgium; 3 Laboratoire de Physiologie et Pharmacologie, Faculty of Medicine, Université libre de Bruxelles (ULB), Brussels, Belgium

**Keywords:** aerobic capacity, muscle, oxygen diffusion, training, vascularisation, VO_2max_

## Abstract

**Background:**

This research demonstrates how two distinct training modalities, high-intensity interval training (HIIT) and moderate-intensity continuous training (MICT), influence oxygen transport dynamics and microvascular remodeling.

**Methods:**

Twenty-five healthy sedentary men and women (median age 21 years) were randomly assigned to HIIT or MICT for 8 weeks. VO_2max_ improvement was assessed in all participants. Non-invasive maximal cardiac output measurements (Q_max_) were performed in 15 participants. Biopsies from vastus lateralis were obtained, cleared and immunolabeled for VE-cadherin and alpha-smooth muscle actin, in 10 subjects, to observe microvasculature architecture. A computational hemodynamic model was constructed to estimate muscle flow dynamics.

**Results:**

VO_2max_ and Q_max_ increased significantly in both training groups, with a greater improvement for VO_2max_ in HIIT that was accompanied by a significant increase in capillaries pericyte coverage. No formation of new capillaries nor anastomoses (angiogenesis) was detected in either group. Modelisation estimated higher shear stress during HIIT than MICT and pericyte recruitment was modelized to adapt to shear stress level limiting excessive capillary dilation.

**Conclusion:**

HIIT induces superior improvements in VO_2max_ and distinct microvascular structural adaptations rather than angiogenesis. HIIT is thought to induce protective capillary adaptation, limiting dilation during maximal effort and improving oxygen diffusion.

**Clinical trial registration:**

https://clinicaltrials.gov/study/NCT07237854, identifier NCT07237854.

## Introduction

Aerobic capacity, indexed by maximal oxygen consumption (VO_2max_), strongly predicts cardiovascular health and all-cause mortality (Bull et al., 2020). Regular exercise provokes repeated perturbations of homeostasis that elicit coordinated central and peripheral adaptations, thereby increasing VO_2max_ (Fiorenza et al., 2020). In this context, exercise training modalities such as moderate-intensity continuous training (MICT) and high-intensity interval training (HIIT) (Myrkos et al., 2023) have been successfully used for decades (Scribbans et al., 2016). MICT is typically performed over a relatively extended period of time between 50% and 70% VO_2max_, corresponding to efforts below or near the first ventilatory threshold (VT1). VT1 reflects an individual’s aerobic capacity and marks a shift in substrate utilization, transitioning from predominantly aerobic pathways to increased engagement of anaerobic metabolism. HIIT, on the other hand, alternates between high-intensity exercise (above the VT1 threshold) and recovery phases. Ongoing debate, however, persists as to whether HIIT yields improvements in VO_2max_ that are similar or superior to those of MICT and the exact mechanism of action remains elusive. Crucially, increases in exercise intensity produce larger hemodynamic forces within the microcirculation (arterioles, capillaries, and venules) of active muscles. Hemodynamic forces, represented by fluid shear stress (FSS) and vessel wall circumferential stretch (Hoier and Hellsten, 2014; Baeyens and Schwartz, 2016; Foote et al., 2022), are sensed by mechanoreceptors located between endothelial cells and vascular smooth muscle cells, triggering intracellular signalling cascades that may drive vascular remodeling (Baeyens et al., 2015; Deng et al., 2021; Bartoli et al., 2022). Because direct *in vivo* quantification of microvascular hemodynamic forces within human skeletal muscle remains technically challenging, computational hemodynamic modeling may provide an important complementary framework for estimating how exercise-induced vascular remodeling influences flow distribution, shear stress, and impact the oxygen transport. The sequential nature and elevated intensities of HIIT may provoke greater, more dynamic, and more frequent hemodynamic stimuli, potentially leading to more pronounced vascular remodeling. According to the Fick principle, VO_2_ is the product of cardiac output and the arteriovenous oxygen difference; thus, improvements in aerobic capacity reflect both central enhancements (e.g., increased cardiac contractility and stroke volume) and peripheral changes (e.g., improved aerobic metabolism and enhanced muscle tissue perfusion) (Skattebo et al., 2020; Balci et al., 2022). This study aims to elucidate how peripheral adaptations, particularly microvascular adaptive remodeling, contribute to improved cardiovascular efficiency and maximal oxygen uptake (VO_2max_) following different exercise regimens. By comparing HIIT and MICT, we sought to characterize intensity-dependent vascular adaptations to training and their potential role in optimizing tissue perfusion, oxygen delivery, and aerobic performance. To further strengthen this mechanistic framework, we incorporated computational hemodynamic modeling to evaluate the theoretical impact that structural vascular remodeling may have on muscle perfusion and oxygen transport. We hypothesized that: 1) both MICT and HIIT would improve VO_2max_ and maximal cardiac output, with HIIT inducing greater improvements in VO_2max_; 2) HIIT would generate stronger hemodynamic stimuli, leading to more pronounced microvascular structural remodeling than MICT; and 3) these peripheral vascular adaptations would enhance muscle oxygen delivery and extraction during maximal exercise.

## Methods

### Study design

**Figure 1:**
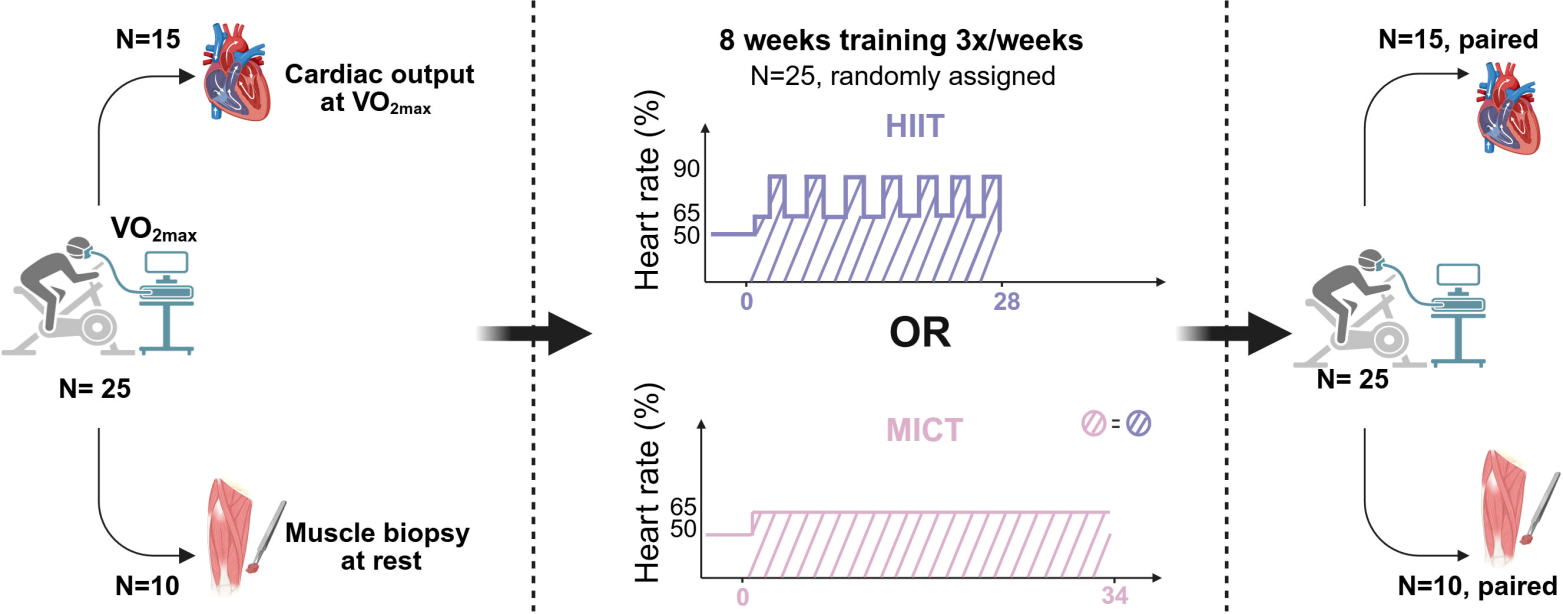
Schematic overview of the study protocol and assessment timeline. Maximal oxygen uptake (VO_2max_) at baseline and following an 8-week training intervention. A subset of 15 participants underwent cardiac output measurements at VO_2max_, while another subset of 10 participants underwent muscle biopsy of the vastus lateralis at rest. Participants were randomly assigned to either a high-intensity interval training (HIIT) or moderate-intensity continuous training (MICT) group. Both training modalities were matched for hemodynamic load, defined as the product of session duration and target intensity, individualized according to each participant’s percentage of maximal heart rate.

This prospective study was conducted between September 2022 and December 2024 within the Research Unit of Rehabilitation Sciences at Université libre de Bruxelles. All participants received detailed information about the study and provided written informed consent, which was approved by the Research Ethics Committee of the Brussels University Hospital (B4062021000227). Inclusion criteria required participants to be between 18 and 40 years old, to have a body mass index (BMI) below 30, and be classified as sedentary. Sedentary status was defined as self-reported physical activity levels below the World Health Organization guidelines, less than 150 minutes of moderate-intensity or 75 minutes of vigorous-intensity exercise per week (Bull et al., 2020). Exclusion criteria included smoking, regular medication use, and any history of cardiovascular, respiratory, or metabolic diseases. The study protocol is illustrated in **Figure 1**. Twenty-five participants were recruited and underwent a cardiopulmonary exercise test (CPET) to determine their maximal oxygen uptake (VO_2max_). Of those 25 subjects, a subgroup of 15 participants completed a second exercise test after 24 hours and following a similar workload increment, with measurement of non-invasive cardiac output at rest and at maximal exercise workload (Q_max_). VO_2max_ and Q_max_ were subsequently used to derive the maximal arteriovenous difference (a-v O_2_ diff max) according to the Fick principle. Another subgroup of 10 participants underwent a resting muscle biopsy of the vastus lateralis (quadriceps). Muscle samples were collected, fixed and processed as detailed below (*Muscle Biopsies* section). All participants were then enrolled in an 8-week training program, 3 times a week, and randomly assigned to either a MICT group or HIIT group. At the end of the 8-week intervention, all baseline assessments were repeated. Cardiac output measurements and muscle biopsy data were used to construct a hemodynamic model (*Hemodynamic model* section) designed to link macro-level cardiovascular adaptations with micro-level vascular changes.

### Training modalities

Cycling-based exercise training was performed under supervision three times per week. Throughout each session, heart rate was continuously monitored with visual feedback to facilitate adherence to prescribed intensity targets. The training workload was adjusted every five sessions, following individualized heart rate zones. Both exercise protocols were designed to elicit an equivalent hemodynamic load, defined as the product of training duration and targeted heart rate. All training sessions (HIIT and MICT) commenced with a standardized 3-minute warm-up at 50% HR_max_. The 28-minute HIIT protocol consisted of seven 2-minute bouts at 90% HR_max_, each separated by 2 minutes of moderate-intensity exercise at 65% HR_max_. The 34-minute MICT protocol involved continuous exercise at 65% HR_max_ for 34 minutes. HR_max_ was determined from the initial cardiopulmonary exercise test (CPET).

### Cardiopulmonary exercise test

CPET was conducted on a cycle ergometer (Ergoselect 100, Ergoline GmbH, Germany) using a one-minute workload increment protocol. Participants were instructed to maintain a pedaling cadence between 60 and 70 revolutions per minute throughout the test. Oxygen consumption (VO_2_), carbon dioxide production (VCO_2_), and ventilation (VE) were measured breath-by-breath via a face mask (COSMED, Rome, Italy). VO_2max_ was defined as the highest VO_2_ value recorded over a 20-second interval at peak exercise. Additional methodological details, including test initiation procedures, criteria for maximal effort, and determination of the first ventilatory threshold, are provided in the **Supplementary Methods** (online appendix).

### Non invasive measure of cardiac output

Cardiac output (Q) was measured non-invasively using the inert gas rebreathing method with the Innocor device (COSMED, Rome, Italy), which operates based on a single-alveolar lung model. Stroke volume was calculated by dividing the cardiac output (Q) by the heart rate (HR). Participants rebreathed a gas mixture containing 0.5% nitrous oxide (NO, blood-soluble) and 0.1% sulfur hexafluoride (SF_6_, blood-insoluble) diluted with ambient air. Pulmonary shunting is minimal in healthy individuals; pulmonary blood flow (PBF) was assumed to be equivalent to Q (PBF ≈ Q). Additional technical details are provided in the **Supplementary Methods** (online appendix).

### Muscle biopsies

**Figure 2:**
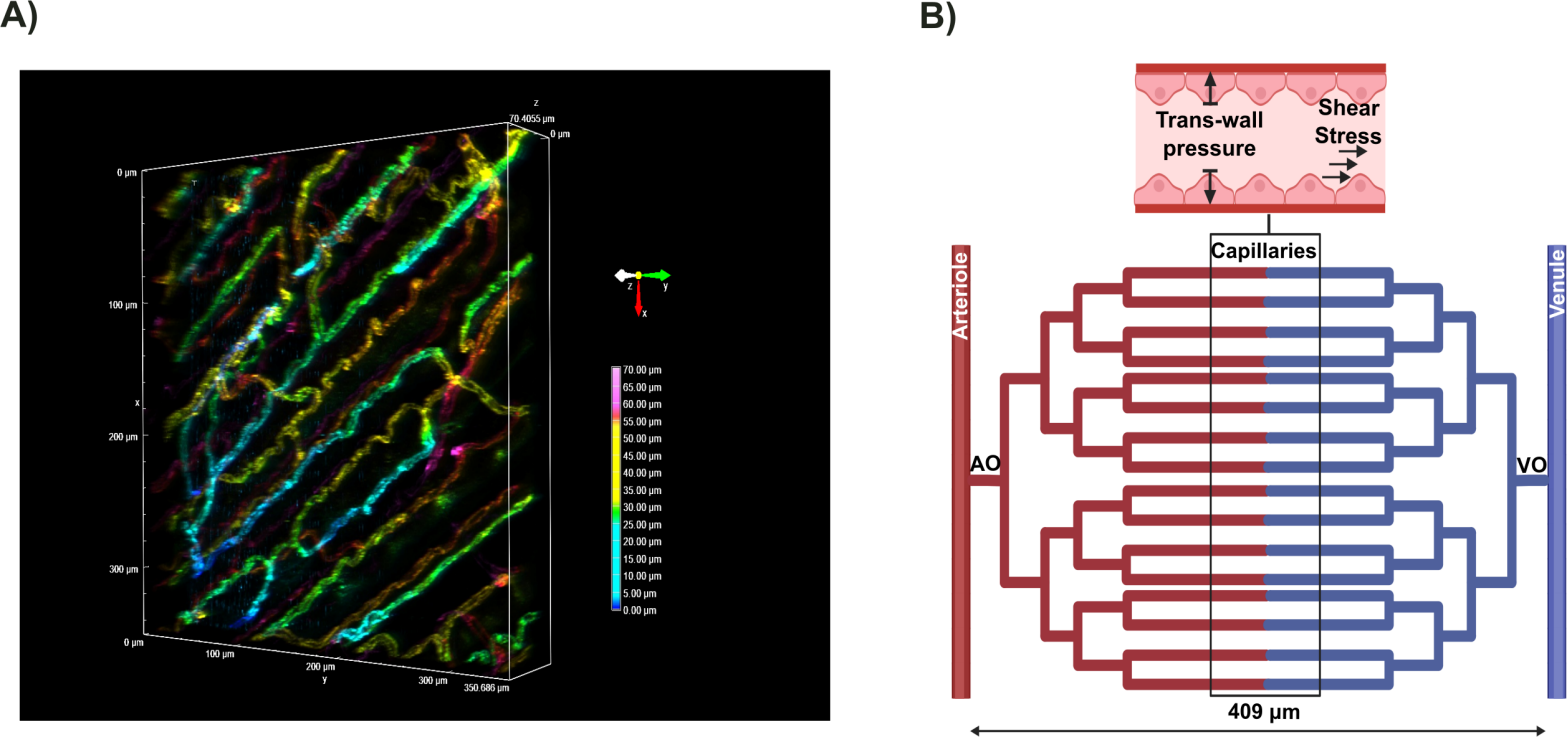
**(A)** represents 3D confocal image showing the capillary network within the muscle biopsy and illustrating the volumetric organization of the microvascular architecture within a defined tissue volume (350 µm × 350 µm × 70 µm). Depth-coded color mapping is directly integrated into the 3D image, where blue indicates the most superficial structures and pink the deepest, providing spatial visualization of capillary distribution throughout tissue depth. **(B)** depicts the microvascular hemodynamic model developed to simulate flow dynamics across a branching capillary network. The structure consists of a single arteriole outlet (AO) bifurcating into five successive generations, culminating in 32 individual capillary segments that reconverge into a single venule outlet (VO). The total length of the model spans 409 mm. Hemodynamic forces were estimated throughout the network under physiologically relevant conditions.

Resting muscle biopsies were obtained from the vastus lateralis of the quadriceps 14 days before the baseline CPET and 5 days after the post-training CPET to minimize any discomfort or pain that could interfere with exercise performance. To avoid sampling scar tissue and ensure anatomical consistency, the first biopsy was performed on the right leg and the second on the left. Muscle samples were processed using the PEGASUS protocol, a modified tissue-clearing technique optimized for immunolabeling and three-dimensional imaging. Vessel inner diameters (intima) were obtained from VE-cadherin staining of the endothelium, whereas external diameters (media) were determined using alpha-smooth muscle actin (alpha-SMA) staining of smooth muscle cells and pericytes. Confocal three dimensional imaging was performed using a Nikon AX R confocal microscope (**Figure 2A**). For each sample, z-stack acquisitions at 10× magnification were stitched together to generate wide-field composites that encompassed the full tissue volume. From these representative regions, areas containing arteriolar, capillary, and venular segments were selected for high-resolution imaging at 20× magnification. Vessel diameters were quantified transversely using NIS-Elements software, based on fluorescence-defined boundaries. Intima diameters were defined by VE-cadherin signal, while media diameters corresponded to α-SMA-positive structures. Measurements were performed independently on each fluorescence channel to ensure layer-specific morphometric accuracy. Capillary density was assessed in two anatomically distinct regions per sample using a volumetric approach that combined z-stack depth with a fixed surface area of 200 × 200 µm, with a mean imaging depth of 70 ± 6 µm at baseline and 83 ± 23 µm following the training intervention. A Full z-axis navigation allowed discrimination of individual vessels throughout tissue depth, minimizing errors related to vessel overlap or superimposition. Additional procedural details regarding biopsy collection, tissue preparation, immunostaining, and clearing steps are provided in the **Supplementary Methods** (online appendix).

### Hemodynamic model

To improve our understanding of hemodynamic forces and how the microvascular system might structurally adapt to these forces, we developed a hemodynamic model representing the microvascular network. The hemodynamic model consisted of five dichotomous branches of the arteriole, resulting in 32 capillaries per arteriole. The venous side of the network consisted of five dual merges, giving one venule per arteriolar (**Figure 2B**).


$$d_{i+1}=2^{-1/2+\epsilon_{1}}d_{i}\;d_{i}=2^{-1/2+\epsilon_{2}}d_{i+1}\;l_{i+1}=2^{-1/3-\gamma_{1}}l_{i}\;l_{i}=2^{-1/3-\gamma_{2}}l_{i+1}$$

(Honig et al., 1977; Huo and Kassab, 2012).

We distinguished three regimes of flows: Fåhræus-Lindqvist regime for vessel with a diameter above 8 µm, lubrication regime for vessels with a diameter below 7 µm and mixed regime for vessels with a diameter between 7 and 8 µm.


$$\begin{array}{c} r-r^{\prime }=0.044r+0.74\frac{{µ}_{s}}{µ}=1+{\left(\frac{r^{\prime }}{r}\right)}^{4}\left(\frac{{µ}_{s}}{{µ}_{b}}-1\right)\frac{µ}{{µ}_{s}}=1+kH_{cap}\frac{H_{cap}}{H}=\sigma^{2}\left[1+\frac{{\left(1-\sigma^{2}\right)}^{2}}{\sigma^{2}\left(2-2\sigma^{2}+\sigma^{2}\frac{{µ}_{s}}{{µ}_{b}}\right)}\right]\sigma =\\ r^{\prime }/rk=\frac{\pi r^{2}}{V_{RBC}}=\left[\frac{\Delta pr^{2}}{8µ\left(u_{0}2q_{0}/r\right)}-h_{RBC}\right]u_{0}=\frac{q_{0}}{\pi r^{2}}{\left(1-\frac{H_{cap}}{H}\right)}^{-1}\frac{2µu_{0}}{r}{\left(\frac{2.123ur}{2q_{0}}\right)}^{3/2} \end{array}$$

(Fung et al., 1981; Secomb et al., 1986; Chebbi, 2015; Al-Khazraji et al., 2016; Lowe, 2019).

The network was then deformed to account for the pressure of blood inside the vessels. For each generation, the final diameter was defined as the diameter providing a force balance between the pressure of the blood on the internal side and the elastic force of the walls and the intramuscular pressure on the external side.


$$r=\frac{P_{i}-P_{ext}r_{0}^{2}}{hE}E=\left(\sum_{i}{\left(\frac{h_{i}}{E_{i}}\right)}^{-1}\right)\left(\sum_{i}h_{i}\right)$$

(Tilton et al., 1979; Sun et al., 1998).

We assumed that the total flow in the muscles was 1 litre per minute at rest. For effort, we considered that the increase in cardiac output translated into an increased blood flow directed specifically toward the active muscles, taking into account that during intense exercise up to 80% of the cardiac output is redistributed to the working muscle (Joyner and Casey, 2015). The flow rate in the network was adjusted accordingly. Finally, to specifically assess the independent and combined effects of angiogenesis and vascular remodeling on network hemodynamics, simulations were performed under the post-training exercise flow conditions previously defined in the model (i.e., assuming that 80% of cardiac output was redistributed to active skeletal muscle). Three vascular architectures were compared. First, the baseline pre-training vascular network was simulated with the addition of 12.5% capillary angiogenesis to isolate the specific hemodynamic contribution of angiogenesis alone. This magnitude was selected based on its proximity to the ~15% capillary increase reported following aerobic exercise training (Liu et al., 2022a). Second, the experimentally derived post-training remodeled vascular network was simulated to evaluate the isolated effect of exercise-induced vascular remodeling. Third, the remodeled post-training network was combined with an additional 12.5% capillary angiogenesis to assess the cumulative effects of both remodeling and angiogenic expansion. This approach was designed to distinguish the relative contributions of angiogenesis and vascular remodeling to pressure-drop regulation under equivalent exercise flow demand. As a static experimental model, these simulations did not incorporate dynamic physiological mechanisms such as acute vasodilation, muscle contraction-induced vascular compression, enhanced cardiac pumping function, or skeletal muscle pump-mediated venous return. Across all simulations, vessel lengths were preserved so that pressure-drop differences specifically reflected network configuration and capillary expansion rather than additional geometric modifications. The length and diameter ratios between each vessel generation, the detailed descriptions of the three flow regimes, and the elastic vessel model are provided in the **Supplementary Methods** (online appendix).

### Statistical analysis

All statistical analyses were performed using Jamovi (version 2.3.28). Data normality was assessed for each variable using the Shapiro-Wilk test. Results are expressed as mean ± standard deviation when normality was confirmed, or as median (interquartile range 25–75) when the distribution was non-normal. Paired Student’s t-tests were used to compare baseline and post-training (8 weeks) values. Two-way ANOVA was conducted with time and group as factors, and variance homogeneity was confirmed prior to analysis. Tukey’s *post hoc* test was applied when the ANOVA indicated a statistically significant effect. Statistical significance was accepted at p< 0.05.

## Results

Twenty-five young, healthy, and sedentary individuals completed the 8-week training intervention (median age: 21 years; 15 women and 10 men), with 13 performing MICT and 12 HIIT. No significant differences were observed in baseline characteristics between the groups (**Table 1**). All participants who completed the intervention demonstrated full adherence to the prescribed training protocol. Aerobic capacity (VO_2max_) was similar between groups at baseline. As expected, VO_2max_ increased in both groups following the training intervention, with a mean improvement of 4 ± 2 ml·kg^-^¹·min^-^¹ in the MICT group and 8 ± 5 ml·kg^-^¹·min^-^¹ in the HIIT group. The increase in VO_2max_ was significantly greater in the HIIT group compared to MICT (p = 0.024). Consistently, maximal workload improved by 23 ± 13 watts in the MICT group and by 40 ± 18 watts in the HIIT group with a higher increase in HIIT (p=0.014). Both VO_2_ and workload at the first ventilatory threshold (VT1) showed significant improvements post-intervention in the MICT (p=0.032 and p=0.019, respectively) and HIIT groups (p<0.001 and p=0.007, respectively). These outcomes are summarized in **Table 2**.

**Table 1:** Descriptive characteristics of participants.

Characteristic	MICT (n = 13)	HIIT (n = 12)	p-value
Gender (F/M)	9/4	6/6	0.347
Age (years)	21 (20 – 25)	22 (20 – 27)	0.722
Weight (kg)	70± 10	70 ± 11	0.896
Height (cm)	170 ± 12	171 ± 9	0.842
BMI (kg/m²)	24.5 ± 4.1	23.8 ± 2.2	0.595

BMI, body mass index. Statistics: Normality tests were performed by Shapiro-Wilk test. Student’s t-test was applied for normally distributed data (mean ± SD) and Mann-Whitney U test for non-parametric data (median (25th -75th percentile).

**Table 2:** CPET data at before and following 8 weeks of training.

CPET values	MICT (N = 13)			HIIT (N = 12)			
	Baseline	8 weeks	Time	Baseline	8 weeks	Time	Time* group
Rest							
VO2 (L. min^-^¹)	0.42 ± 0.07	0.44 ± 0.12	0.582	0.39 ± 0.08	0.42 ± 0.07	0.091	0.278
VO2 (mL·kg^-^¹·min^-^¹)	6.1 ± 0.9	6.4 ± 1.4	0.532	5.6 ± 1.1	5.9 ± 0.8	0.145	0.167
HR (beats·min^-^¹)	93 ± 13	85 ± 13	0.082	84 ± 10	85 ± 13	0.743	0.113
VT1							
VO2 (L. min^-^¹)	1.75 ± 0.35	1.94 ± 0.51	0.052	1.82 ± 0.44	2.23 ± 0.42	<0.001	0.038
VO2 (mL·kg^-^¹·min^-^¹)	25 ± 4	28 ± 6	0.032	27 ± 6	31 ± 4	<0.001	0.538
Workload (Watt)	111 ± 27	129 ± 43	0.019	123 ± 43	149 ± 29	0.007	0.451
HR (beats·min^-^¹)	148 ± 19	150 ± 13	0.467	137 ± 24	144 ± 20	0.279	0.526
Maximal							
VO2 (L. min^-^¹)	2.61 ± 0.64	2.92 ± 0.75	0.006	2.56 ± 0.45	3.18± 0.75	<0.001	0.022
VO2 (mL·kg^-^¹·min^-^¹)	37 ± 7	41 ± 9	0.003	38 ± 6	46 ± 9	<0.001	0.024
Workload (Watt)	187 ± 49	210 ± 57	<0.001	198 ± 44	237 ± 51	<0.001	0.014
HR (beats·min^-^¹)	184 ± 11	185 ± 11	0.567	177 ± 13	178 ± 16	0.585	0.978
RER	1.17 ± 0.07	1.16 ± 0.06	0.729	1.17 ± 0.08	1.16 ± 0.09	0.775	0.862

VO2, Oxygen uptake, HR,Heart rate, VT1, first ventilatory threshold, RER, Respiratory Exchange Ratio, Baseline: before 8-weeks training, 8 weeks: After 8-weeks training. Statistics: Normality was tested with a Shapiro-Wilk test. For pairwise comparisons, Student’s t-tests were used. ANOVA tests were performed to compare groups and Tukey post hoc tests were applied. Results are presented as mean ± SD.

### Non-invasive determination of cardiac output

**Figure 3:**
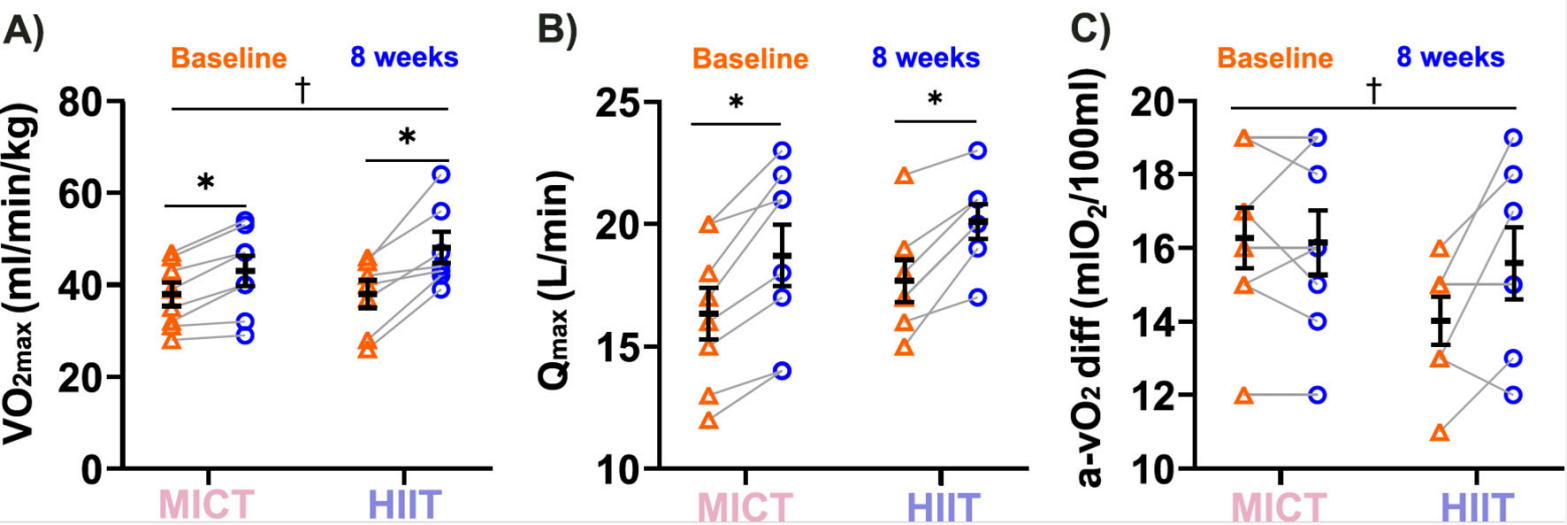
Cardiac output and oxygen transport adaptations following 8 weeks of MICT or HIIT (N = 15). **(A)** illustrates the evolution of VO_2max_ across the training period. Both MICT and HIIT groups demonstrated significant improvements post-intervention; however, the magnitude of increase was more pronounced in the HIIT cohort. **(B)** depicts changes in cardiac output measured at VO_2max_. Both training modalities elicited comparable increases in peak cardiac output. **(C)** presents the maximal arteriovenous oxygen difference (a-vO_2_ diff), calculated using the Fick principle (VO_2_/Q). The HIIT group demonstrated a trend toward greater enhancement in a-vO_2_ diff compared to MICT, with a distinct temporal evolution between groups. Orange triangles represent baseline data; blue circles indicate post-training. Statistics: Normality tests were tested with a Shapiro-Wilk test. ANOVA tests were performed to compare groups, when significant, Tukey post hoc tests were applied. For pairwise comparisons, Student’s t-tests were used. Results are presented as mean ± SD. * denotes a significant difference between pre- and post-training; † indicates a significant difference between time*groups.

Out of the 25 participants, 15 performed non-invasive cardiac output (Q) measurements (8 assigned to MICT and 7 to HIIT). At peak exercise, Q_max_ increased similarly in both groups after 8 weeks of exercise training: from 16 ± 3 L/min to 19 ± 4 L/min (+ 13 ± 7%, p=0.002) in MICT and from 18 ± 3 L/min to 21 ± 2 L/min (+ 15 ± 9%, p=0.001) in HIIT. In parallel, stroke volume increased from 88 ± 13 to 101 ± 17mL (+ 15 ± 8%, p=0.01) in the MICT group and from 100 ± 15 to 119 ± 16mL (+ 19 ± 13%, p<0.001) in the HIIT group. No modification of maximal heart rate was observed. Within this subgroup, VO_2max_ also increased, with a greater improvement in the HIIT group than in the MICT group (p = 0.049). Calculated peripheral O_2_ extraction remained unchanged in the MICT group (16 ± 3 to 16 ± 2 mlO_2_/100ml, p=0.984), while a tendency toward an increase was observed in the HIIT group (14 ± 2 to 16 ± 3 mlO_2_/100ml, p=0.066). However, a significant group-by-time interaction was detected (p = 0.041), suggesting a differential adaptation in peripheral oxygen extraction between training modalities. Q_max_, VO_2max_ and a-vO_2_ diff comparison are presented in **Figure 3**.

### Resting muscle biopsies outcomes

**Figure 4:**
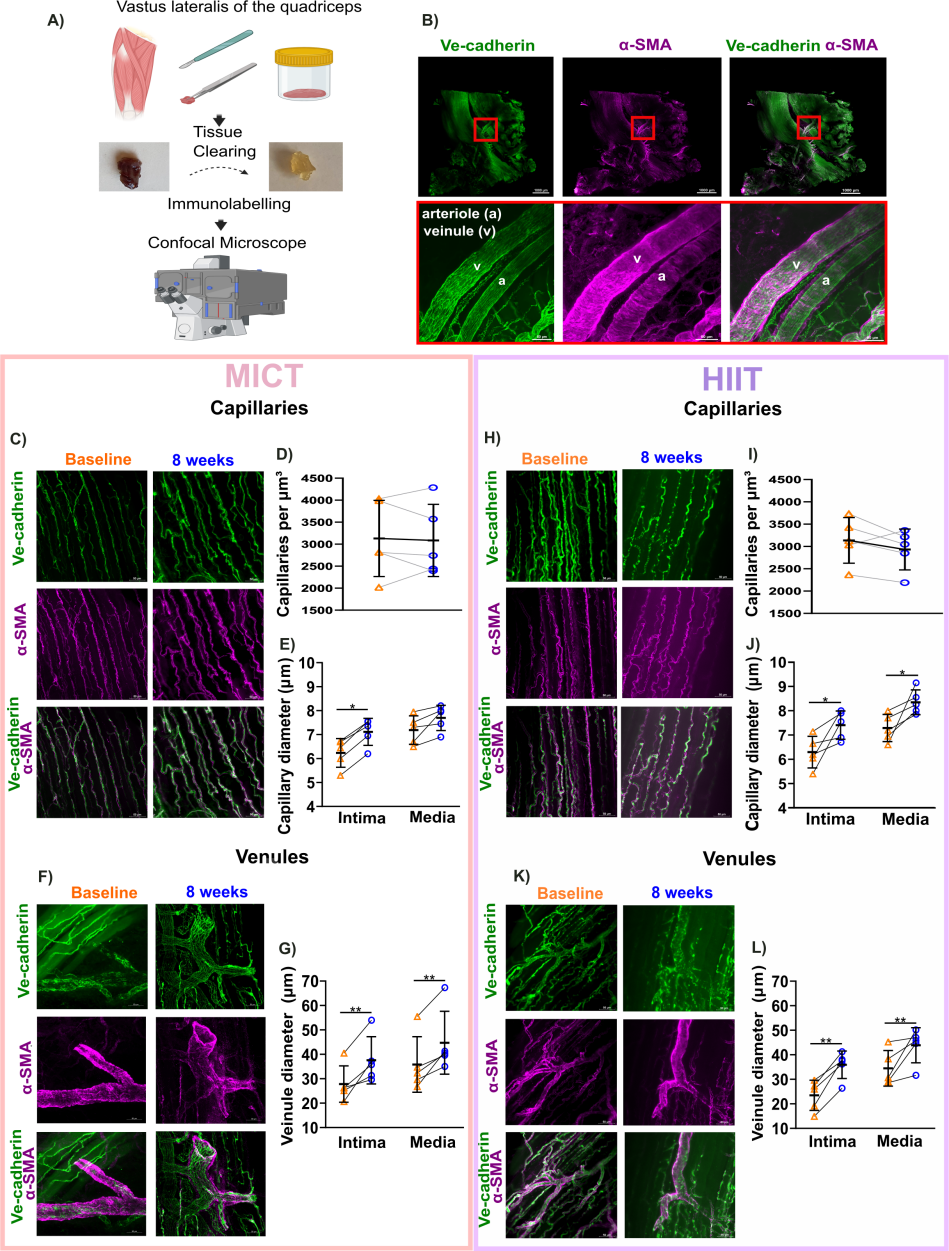
This figure presents the methodological workflow for muscle biopsy processing and image acquisition, as well as the vascular remodeling observed in response to exercise training. **(A)** Schematic overview of the biopsy protocol performed in 10 participants. Samples were collected under local anesthesia from the vastus lateralis using a scalpel, then cleared and immunolabeled with VE-cadherin to visualize endothelial cells and withd α-smooth muscle actin (α-SMA) to identify smooth muscle cells and pericytes. Image acquisition was performed using a Nikon AX R confocal microscope. **(B)** Overview of an entire muscle sample (scale bar: 1000 µm; objective: Apo 10×), with the red box indicating the region of interest. This region contains a venule and an arteriole, distinguishable by their structural features: venules exhibit a loose endothelial architecture with disorganized smooth muscle coverage, whereas arterioles display tightly arranged endothelial cells and concentric smooth muscle rings (scale bar: 50 µm; objective: Apo 20×). **(C–G)** correspond to the MICT group and **(H–L)** to the HIIT group. **(C, H)** represent the visualization of the capillary network before and after 8 weeks of training while **(F, K)** represent the visualization of a venule before and after 8 weeks of training (scale bar: 50 µm; objective: Apo 20×). **(D , I)** is the quantification of capillary network, both group didn’t succeed to increased the number of capillaries after training. **(E, J)** quantify capillary diameter changes at baseline and 8 weeks after training, measured at the intima (VE-cadherin) and media (α-SMA) levels. Both groups exhibited significant dilation of the capillary intimal diameter following training; however, only the HIIT group **(J)** demonstrated a concomitant increase in pericyte coverage. **(G, L)** demonstrate venular remodeling in both groups, with increased intima diameter and enhanced smooth muscle cell coverage. Orange triangles represent baseline data; blue circles indicate post-training. Statistics: Normality tests were performed by Shapiro-Wilk test. For pairwise comparisons, Student’s t-tests were used. Results are presented as mean ± SD. * denotes a significant difference between pre- and post-training.

In a subgroup of 10 participants equally divided between MICT (n=5) and HIIT (n=5), we performed vastus lateralis muscle biopsies before and after an 8-week training regimen. Arteriolar intima diameters exhibited non-significant enlargement from 20±3µm at baseline to 26 ± 4µm post-training in the MICT group (p=0.054), and from 22 ± 5µm to 27 ± 5µm in the HIIT group (p=0.171). Similarly, media diameters of arterioles showed a comparable dynamic, from 30 ± 4µm to 35 ± 7µm in MICT (p=0.181), and from 31 ± 9µm to 41 ± 12µm in HIIT (p=0.147). Capillary intima diameters increased following training, from 6.2 ± 0.6µm to 7.2 ± 0.5µm in MICT (p = 0.001) and from 6.3 ± 0.7µm to 7.4 ± 0.5µm in HIIT (p = 0.045). The media diameters of capillaries (representing pericytes coverage) showed no significant increase, from 7.1 ± 0.6µm to 7.7 ± 0.5µm in MICT (p = 0.06), and an eventual increase from 7.3 ± 0.6µm to 8.3 ± 0.5µm in HIIT (p = 0.047) (**Figures 4E, J**). No pro-angiogenic response was detected in both group, as three-dimensional analyses revealed no measurable expansion of the capillary network across conditions, including neither increased capillary number along vessel length nor increased anastomotic connections between capillaries. In the MICT group, mean capillary density was 3135 capillaries/mm³ at baseline and 2931 capillaries/mm³ post-training (p=0.231). Similarly, the HIIT group exhibited values of 3131 capillaries/mm³ at baseline and 3086 capillaries/mm³ following the intervention (p=0.805) (**Figures 4D, I**). Statistical analysis confirmed the absence of significant differences over time between groups (p = 0.499). Venular internal diameters demonstrated a pronounced increase, from 28 ± 8µm to 38 ± 10µm in MICT (p = 0.015), and from 23 ± 6µm to 36 ± 6µm in HIIT (p = 0.003). Correspondingly, venular external diameters expanded, from 36 ± 11µm to 45 ± 13µm in MICT (p = 0.009), and from 32 ± 10µm to 44 ± 7µm in HIIT (p = 0.021) (**Figures 4G, L**).

**Figure 5:**
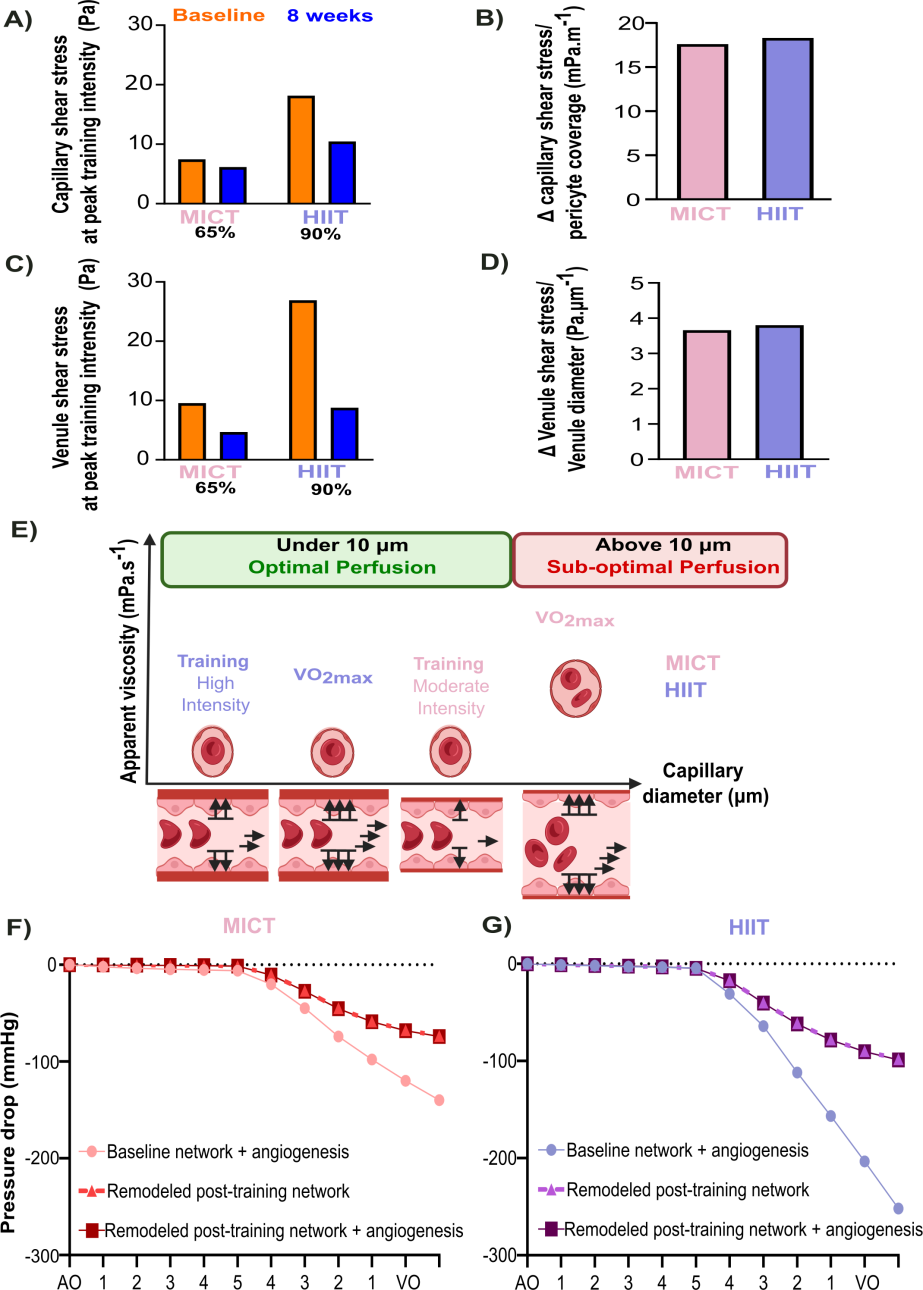
**(A–D)** show all shear stress values at peak training intensity (i.e. 65% HRmax for MICT and 90% HR_max_ for HIIT). **(A)** displays capillary shear stress in both training group. **(B)** shows the ratio between training-induced changes in capillary shear stress and the relative variation in pericyte coverage following the 8-week intervention. **(C)** reports venular shear stress values across MICT and HIIT conditions. **(D)** illustrates the relationship between changes in venular shear stress and corresponding alterations in venular diameter. **(E)** characterizes apparent blood viscosity as a function of vessel diameter, highlighting red blood cell (RBC) morphology and axial positioning in capillaries below and above 10 mm in diameter. The number and orientation of flow vectors reflect the magnitude and directionality of local hemodynamic forces within the capillary bed. **(F, G)** illustrate the simulated pressure drop across the hemodynamic model under post-training maximal cardiac output. For each training condition, MICT in **(F)** and HIIT in **(G)**, pressure drop was calculated in: (1) the baseline vascular network with simulated angiogenesis (+12.5% capillary expansion), (2) the post-training remodeled vascular network, and (3) the post-training remodeled vascular network with additional simulated angiogenesis (+12.5%), allowing comparison of how isolated angiogenesis versus vascular remodeling influenced hemodynamic efficiency under maximal flow conditions.

### Hemodynamic model

Based on estimations from our vascular model capillary shear stress decreased in response to both training modalities after eight weeks. After training, and under training-intensity conditions (i.e., 65% HR_max_ for MICT and 90% HR_max_ for HIIT), capillary shear stress declined: from 7.45 Pa to 6.16 Pa in MICT and from 18.15 Pa to 10.47 Pa in HIIT. This indicates that, under the same conditions of exercise/blood flow intensity, shear stress before training was higher than after (**Figure 5A**). The relative reduction in shear stress during training yielded similar ratios with the increase in pericyte coverage for both modalities: 17.6 MPa·m^-^¹ for MICT and 18.35 MPa·m^-^¹ for HIIT (**Figure 5B**). Similarly to capillaries, venular shear stress decreased from baseline to post-training. Under the same exercise training intensity, venular shear stress dropped from 9.55 Pa to 4.69 Pa with MICT and from 26.90 Pa to 8.79 Pa with HIIT (**Figure 5C**). The ratios between changes in venular shear stress and corresponding alterations in venular diameter were consistent: 3.66 for MICT and 3.80 for HIIT (**Figure 5D**). These ratios reflect the relationship between the increase in venular diameter and the corresponding reduction in venular shear stress during training. Furthermore, we hypothesize that enhanced capillary pericyte coverage under HIIT conditions may contribute to mechanical stabilization of the capillary wall, potentially limiting deformation under elevated shear forces. This is illustrated in **Figure 5E**. Simulations of pressure-drop across distinct vascular architectures revealed marked differences in network hemodynamic efficiency depending on structural configuration. In MICT conditions (**Figure 5F**), the baseline vascular network supplemented with 12.5% angiogenesis exhibited a pressure drop of 140 mmHg, whereas the post-training remodeled vascular network demonstrated a substantially lower pressure drop of 74 mmHg. The addition of 12.5% angiogenesis to the remodeled network did not further alter this response, with pressure drop remaining at 74 mmHg. Similarly, under HIIT conditions (**Figure 5G**), the baseline vascular network supplemented with 12.5% angiogenesis exhibited a pressure drop of 252 mmHg, while the remodeled post-training vascular architecture reduced pressure drop to 98 mmHg. The subsequent addition of 12.5% angiogenesis to the remodeled network again produced negligible additional effect, with pressure drop measured at 99 mmHg.

## Discussion

Our results demonstrate that both MICT and HIIT improved aerobic capacity, with HIIT eliciting greater increases in VO_2max_ despite similar improvements in maximal cardiac output. At the microvascular level, both training modalities were associated with structural remodeling of pre-existing vessels, including capillary dilation and venular enlargement, without detectable capillary network expansion under our experimental conditions. Notably, only HIIT was associated with increased pericyte coverage around capillaries, whereas no such adaptation was observed following MICT, suggesting an intensity-dependent effect on capillary mural cell remodeling. Our hemodynamic model further suggests that these structural adaptations may enhance oxygen diffusion within muscle fibers. Collectively, these findings indicate that early exercise adaptation in young sedentary adults primarily involves remodeling of existing microvascular architecture rather than measurable angiogenesis.

### Aerobic capacity

Our results demonstrate that both MICT and HIIT induce significant improvements in VO_2max_ over an eight-week intervention in sedentary young adults. In parallel, the magnitude of improvement in VO_2max_ was significantly greater following HIIT, reinforcing the growing body of evidence supporting its superior efficacy in enhancing aerobic capacity (Scribbans et al., 2016; Crowley et al., 2022). This differential effect appears to be particularly pronounced in healthy individuals aged 14 to 45 years (Guo et al., 2023) and influenced by baseline fitness level, while remaining largely independent of sex (Lock et al., 2024). These effects were also observed by Milanović et al. (2015) reporting greater improvement in VO_2max_ following HIIT, compared to MICT under matched training volumes, suggesting that training intensity is a key driver of aerobic adaptations. It is noteworthy that the magnitude of VO_2max_ improvement observed in our HIIT group exceeds that reported in the meta-analysis of Scribbans et al. (2016) that found a mean increase in VO_2max_ of 4.9 ± 1.4 ml·kg^-^¹·min^-^¹ for MICT versus 5.5 ± 1.2 ml·kg^-^¹·min^-^¹ for HIIT what could partly be explained by the substantial heterogeneity in HIIT protocols across studies. Manipulation of the FITT principle which refers to frequency, intensity, time and type, has indeed emerged as a critical determinant of both acute and chronic, physiological and metabolic responses to interval training (Stavrinou et al., 2025). Wen et al. (2019) accordingly reported that protocols incorporating intervals of at least 2 minutes in duration, combined with a cumulative high-intensity workload of no less than 15 minutes per session over a period of 4 to 12 weeks, consistently produced greater improvements in aerobic fitness compared with MICT. Similarly, Guo et al. (2023) showed also that training with high intensity intervals lasting between 1 and 3 minutes, performed three times per week for a minimum of six weeks, optimize cardiorespiratory adaptations. The superior efficacy of longer HIIT bouts likely stems from sustained near-maximal intensity, enhancing central and peripheral cardiovascular stimuli (Balci et al., 2022). The greater VO_2max_ improvement observed in our study may reflect our protocol’s specific design of 14 minutes cumulative training of 2 minutes intervals at 90% of HR_max_.

### Peripheral oxygen extraction

In our subgroup of 15 participants VO_2max_ improved across both training modalities with a parallel and similar increase in Q_max_. These observations are in line with VO_2max_ increase being primarily related to an increase in maximal oxygen transport (i.e. Q_max_) to the exercising muscle, as described in several meta-analyses (Montero et al., 2015a; Astorino et al., 2022). However, compared to the MICT group, VO_2max_ increased slightly more in the HIIT group, suggesting a better oxygen extraction at maximal exercise. Although the arteriovenous oxygen difference (a-vO_2_ diff) remained generally stable among our participants, we observed a non-significant trend toward a greater increase in the HIIT group (14 ± 2 to 16 ± 3 mlO_2_/100ml, p=0.66) that was accompanied by a temporally divergent trajectory between training modalities (p=0.041). Oxygen extraction is considered to result from a combination of factors, including an intensified Bohr effect (Mairbäurl, 2013), prolonged erythrocyte transit time, increased capillary density (Hellsten and Gliemann, 2024), optimized vascular recruitment and flow distribution (Poole et al., 2020), and greater oxidative capacity of muscle tissue (Zhang and Gao, 2021). In that context, greater exercise intensity during HIIT appears to amplify muscle intracellular signaling cascades, resulting in a superior increase in mitochondrial content compared to work-match MICT protocol (MacInnis and Gibala, 2017). Moreover, repeated exposure to elevated hemodynamic forces, which characterizes HIIT, appears to induce superior vascular adaptations compared to traditional endurance training (Green et al., 2017). A meta-analysis conducted by Montero et al. (2015b) reported no significant improvement in a-vO_2_ diff following aerobic training (mixing MICT and HIIT protocols), although studies with longer duration or higher load of training induced a higher a-vO_2_ diff. However, as highlighted by Poole (Poole et al., 2016), these reports should be interpreted with caution as of some of the studies included in this meta-analysis were subject to methodological limitations related to a limited use of catheter-based measurements and potential inaccuracies in VO_2max_ and Q_max_ estimations. In contrast, a more recent meta-analysis by Astorino et al. (2022) suggests that aerobic training, particularly when involving high-intensity intervals, can elicit modest but significant increases in a-vO_2_ diff. Finally, our results tend to confirm HIIT superiority in improving a-vO_2_ diff at maximal exercise that may stem from vascular remodeling processes, which could facilitate more efficient oxygen extraction as explained below.

### Vascular remodeling

While macrovascular adaptations to exercise have been extensively documented, data regarding microcirculatory remodeling in humans remain limited. Thijssen et al. (2011) demonstrated a dose-dependent improvement in femoral artery flow-mediated dilation (FMD) and reduced arterial wall thickness following aerobic training. Meta-analyses further support a positive correlation between exercise intensity and endothelial function (Ashor et al., 2015), with HIIT yielding greater macrovascular benefits than MICT (Ramos et al., 2015). In contrast, little to no data exist describing structural adaptations at the microvascular level (arteriolar, capillary, and venular diameters), as much of the current understanding stems from animal models, and *in vivo* technical challenges restrict investigation within human quadriceps muscle. To our knowledge, our study is the first to assess microcirculation diameters and angiogenic adaptations in human skeletal muscle using advanced tissue clearing techniques combined with high-resolution confocal microscopy, which enables the three-dimensional visualization of entire human muscle samples, along with micron-level precision for measuring diameters from microcirculation and conducting longitudinal capillary quantification. Direct comparison with previous literature is therefore limited, as most studies on exercise-induced angiogenesis have relied on conventional two-dimensional histological sections with transverse slices stained with immunomarkers to estimate capillary density (capillaries per mm²) or capillary-to-fiber ratios. Other investigations have focused on the expression of angiogenesis-related biomarkers, especially the vascular endothelial growth factor (VEGF) (Jensen et al., 2004; Hoier and Hellsten, 2014). From those studies, capillary density is estimated to range between ~300 and 600 capillaries per mm², depending on training status (Hellsten and Nyberg, 2015) with potential increase of 49.7 capillaries/mm² following endurance training (~15% improvement (Liu et al., 2022a)), and that exercise intensity is a key modulator of an angiogenetic response (Liu et al., 2022b). Interestingly, numerous studies (Ross et al., 2023) (Haas and Nwadozi, 2015) (Hendrickse and Degens, 2019) have identified shear stress as a key biomechanical stimulus for angiogenesis. While extensive literature supports exercise-induced angiogenic responses in human skeletal muscle, our study did not detect evidence of measurable capillary network expansion under our specific conditions. Using advanced tissue clearing, high-resolution confocal microscopy, and immunostaining, we achieved three-dimensional longitudinal visualization of vascular architecture with micron-level precision. This approach provides greater anatomical fidelity than conventional two-dimensional cross-sectional methods by enabling direct assessment of vessel continuity, branching, and anastomotic organization throughout tissue depth. In contrast, two-dimensional transverse sections may overestimate capillary number by counting vessel bifurcations or anastomoses as separate structures, potentially contributing to higher estimates of angiogenesis in previous studies. Moreover, we observed a general increase in capillary diameters after training, suggesting pre-existing capillaries dilatation rather than the formation of entirely new ones. The increased vascular caliber likely improves the detectability of pre-existing capillaries that existed before training but fell below the resolution threshold. Collectively, our findings indicate that, under our experimental conditions, microvascular remodeling was the predominant observable adaptation, without evidence of capillary angiogenesis after 8 weeks of training. Our participants were previously sedentary, and their vascular system was therefore likely more sensitive to exercise-induced hemodynamic stimuli, permitting relatively rapid early remodeling, leaving angiogenesis for a later adaptation. In contrast, trained individuals or athletes often already exhibit substantial cardiovascular and peripheral adaptations (Mølmen et al., 2025). According to the shear stress set-point concept, repeated training may shift or expand the range of mechanical stimuli perceived as physiological, such that a habitual exercise stimulus may no longer be sufficient to induce further vascular remodeling (Maufroy et al., 2026). Consequently, greater, more intense, or more specialized exercise stimuli may be required to exceed this expanded set-point and provoke additional vascular adaptations. Importantly, our computational hemodynamic model supports the hypothesis that remodeling of the baseline vascular network provides more effective pressure-drop regulation than physiologically relevant angiogenesis alone. Interrestingly, angiogenesis in combination with remodeling promotes effective pressure drop similar than when solely remodeling operates. However, these interpretation should be interpreted within the context of the model’s experimental and static design. Because dynamic physiological mechanisms could not be incorporated at the microvascular level, absolute pressure-drop values should be interpreted with caution. Specifically, this framework does not account for acute exercise-related factors such as muscle contraction-induced vascular compression, transient vasomotor responses, enhanced venous return through the skeletal muscle pump, or broader dynamic cardiovascular adjustments during exercise. Consequently, while the model provides valuable comparative insight into the relative structural hemodynamic effects of remodeling versus angiogenesis, it does not fully reproduce the complex dynamic physiology of exercising skeletal muscle.

### Modelisation and interpretation

In humans at rest, typical shear stress values range between 0.5 and 5 Pa (Baeyens and Schwartz, 2016). Numerous studies (Baeyens et al., 2016; Green et al., 2017; Deng et al., 2021) have introduced the concept of a “set point”, whereby endothelial cells drive vascular remodeling, adapting vessel diameter and luminal area to maintain shear stress within an optimal physiological range. A particularly compelling insight from the work of Baeyens and Schwartz (2016). is that the set point may differ across vessel types. In their study, comparison of endothelial cells from veins and lymphatic vessels highlighted the functional heterogeneity of endothelial responses. In our exercise-based model, structural adaptation following blood flow elevation and increased shear stress stimulated both capillaries and venules. Since the biopsies were obtained at rest, the adaptations observed in the capillary and venular segments represent permanent structural changes, in addition to functional vessel adaptation. However, a more pronounced arteriolar vasoreactivity, as evidenced by improved FMD in response to training (Ashor et al., 2015), might still exist without structural modifications. Supporting this interpretation, an experimental investigation of the rat mesenteric microvascular network reported that a ligature-induced rise in local pressure produced no diameter changes in small arterioles (10–20 µm), whereas larger arterioles (20–30 µm and >30 µm) exhibited clear structural adaptations. In contrast, venular segments displayed diameter alterations across all size classes (Van Gieson et al., 2003). Furthermore, our study demonstrated a significant increase in pericyte coverage at the capillary level following HIIT, whereas MICT did not produce a similar effect. This divergent pattern suggests that HIIT may trigger the activation of protective mechanisms to regulate microvascular architecture. Although both training modalities were matched for total exercise volume, exercise intensity appears to be a key determinant of the nature and magnitude of vascular remodeling. Within the shear stress set-point framework, both MICT and HIIT stimulated structural adaptation through elevations in hemodynamic forces (Maufroy et al., 2026). However, the alternance of higher stimulation of HIIT may have more frequently and overall more intensively exceeded the physiological threshold promoting more frequent mechanical stimulation of greater magnitude than in MICT. This may therefore explain why both protocols induced remodeling of pre-existing vessels, while only HIIT promoted additional pericyte coverage. Furthermore, high-intensity intervals performed during HIIT are more representative of VO_2max_ conditions and may promote microvascular adaptations optimized for maximal exercise demands. We hypothesize that the increased pericyte coverage observed under HIIT may help stabilize the microvessel structure in response to high mechanical stimuli, thereby preserving an optimal diameter for oxygen exchange *(***Figure 3F***)*. From a rheological standpoint, vessel diameter has profound effects on blood viscosity and the spatial dynamics of red blood cells (RBCs) (Bateman et al., 2003). In vessels larger than 10 µm, multiple RBCs can travel side-by-side, interact and shift away from the vessel center, leading to peripheral displacement and increased apparent viscosity. In small vessels (diameter<10 µm), red blood cells (6–8 µm) deform and align in single file, moving centrally with a thin plasma layer separating them from the vessel wall, a behaviour described by the Fåhræus–Lindqvist effect. This microvascular flow configuration is especially favourable for oxygen diffusion, as it increases the surface area available for RBC gas exchange making microvascular oxygen transport tightly related to capillary geometry and RBC alignment. Studies have shown indeed that when RBCs travel centrally and in single file, oxygen extraction becomes more efficient due to minimized diffusion barriers (Bateman et al., 2003). We propose that the increased pericyte coverage observed in HIIT may help stabilize capillary diameter against pressure-induced deformation, thereby preserving this optimal flow configuration. This interpretation is further supported by our post-training analysis of the shear stress-to-pericyte coverage ratio yielded comparable ratios in both protocols inducing that pericyte coverage increases proportionally to the shear stress experienced during training. Such adaptation likely acts as a biomechanical safeguard, limiting excessive capillary deformation and helping maintain an optimal vessel diameter for oxygen exchange at specific training intensities. When extrapolated to maximal effort, this mechanism may carry important functional implications as when blood flow and shear stress surge, vessels conditioned through MICT, (i.e. lower shear stress) will exceed their tolerance, leading to over-dilation and reduced oxygen diffusing ability. In contrast, HIIT-trained vessels, conditioned to higher shear stress, are better equipped to withstand peak hemodynamic loads and preserve optimal flow geometry at maximal exercise intensity. Interestingly, veins adapted in accordance to the set point theory (Baeyens et al., 2015) and consistent with a homeostatic response to shear stress elevation, modulating luminal diameter to stabilize local hemodynamic stress. Indeed diameter increase following training was proportionate to hemodynamic forces increases in MICT or HIIT (**Figures 3D, E**). To note, Tiezzi et al. (2022) mentioned shear stresses in veins ranging from 0.1 to 0.6 Pa in healthy individuals. In our cohort, resting shear stress levels decreased after training from 0.9 to 0.7 Pa in the MICT group and from 1.3 to 0.8 Pa in the HIIT group. These higher values are probably explained by the smaller diameters of venules compared to veins. Finally, no evidence of angiogenesis was observed following our exercise training. These findings may appear to contrast with the prevailing paradigm that exercise commonly induces angiogenesis. However, under our specific experimental conditions, our hemodynamic simulations suggest that the only remodeling of the pre-existing vascular architecture markedly enhanced hemodynamic efficiency. In both MICT and HIIT, the structurally remodeled network under post-training flow conditions substantially reduced pressure drop, while simulated physiologically relevant angiogenesis (12.5%) had negligible additional impact. Rather than excluding angiogenesis as a potential longer-term adaptation, these results suggest that, during early training in young sedentary adults, microvascular adaptation may primarily favor mechanotransduction-driven remodeling of existing vessels as a more efficient strategy to optimize perfusion and oxygen delivery. Our findings therefore support the concept that early exercise-induced vascular adaptation may be predominantly governed by structural optimization of the existing microvascular network before substantial angiogenic expansion becomes necessary.

### Limitation

Vessel diameters were measured at rest, as it is impossible to fix perfused human biopsies. Second, blood samples were not collected, preventing direct assessment of hemoglobin concentration and limiting the precision of rheological estimations. Future studies could strengthen both functional and vascular remodeling interpretation by incorporating complementary approaches such as near-infrared spectroscopy (NIRS) to assess local muscle oxygenation and hemodynamic responses during exercise (Jones et al., 2016). Third, cardiac output measurements and muscle biopsy data were collected from two distinct subject subgroups. Although both populations were matched for age, training status, and cardiovascular fitness, the absence of direct overlap introduces a methodological constraint. Consequently, while the findings offer valuable insight into microvessel behaviour, they may lack the granularity required to establish individual-level associations between flow dynamics and vascular remodeling.

In conclusion, our study demonstrates that high-intensity interval training elicits a more pronounced increase in VO_2max_ compared to moderate-intensity continuous training, despite both protocols inducing similar enhancements in maximal cardiac output. We observed training intensity-dependent remodeling in pericyte capillaries, with no evidence of angiogenesis, to maintain optimal diameter and optimize muscle perfusion and oxygen extraction under the highest metabolic hemodynamic stress.

## Data Availability

The original contributions presented in the study are included in the article/**Supplementary Material**. Further inquiries can be directed to the corresponding author.
