# Supplementary material for: Vascular remodeling enhances high-flow muscle oxygen delivery following aerobic exercise training
Source: Front Physiol. 2026 Jul 1;17:1840439. doi: 10.3389/fphys.2026.1840439 (PMC13371106; doi:10.3389/fphys.2026.1840439)
Supplement: Supplementary file 1 [file SupplementaryFile1.docx]

Supplemental Methods

**Cardiopulmonary exercise test (CPET)**

The CPET protocol began with a 3-minute warm-up phase, followed by workload increments ranging from 15 to 25 watts, adjusted according to participants’ estimated physical fitness to ensure a total test duration between 8 and 12 minutes. Cardiac activity was continuously monitored using standard exercise electrocardiography (Medcare, Medisoft, Belgium), heart rate (HR) was recorded via a chest strap monitor (Garmin), and blood pressure was measured at the end of each stage using the ergometer’s integrated sphygmomanometer. The test was considered maximal when participants voluntarily ceased pedalling and at least two of the following criteria were met: respiratory exchange ratio > 1.15; VO₂ increase < 100 mL·min⁻¹ despite workload progression or HR exceeding 90% of age-predicted maximum. The anaerobic threshold was determined using the V-slope method, corroborated by the evolution of VE/VO₂ [1].

**Non invasive measure of cardiac ouput**

During the measurement, participants sat on a cycle ergometer wearing a nose clip and a mouthpiece fitted with a bacterial filter. The gas mixture was rebreathed from a reservoir bag, and the system adjusted rebreathing parameters based on each participant’s real-time respiratory measurements. Each measurement consisted of 5–8 rebreathing cycles, with the first 2–3 breaths excluded from analysis to account for incomplete gas mixing. The system corrected gas volume using end-tidal SF₆ concentration, and regression slopes were recalculated according to the formulas provided in the Innocor user manual to optimize measurement accuracy. Pulmonary blood flow was then calculated using the Innocor software, based on the logarithmic decay of alveolar nitrous oxide concentration over time.

**Muscle Biopsies protocol**

Upon arrival, participants were positioned in a supine position with their legs inactive for 10 minutes to ensure a standardized resting state. Under local anaesthesia with lidocaine, a 1 cm × 2 cm elliptical skin incision was made on the lateral aspect of the lower third of the thigh. The incision was extended through the skin, subcutaneous tissue, and muscle fascia to access the muscle. A muscle sample of approximately 1 cm³ was then extracted. Immediately after collection, the tissue sample was rinsed in phosphate-buffered saline (PBS) and subsequently immersed in a formalin fixative solution (PBS/formol 10%, pH7.4) at 4°C for 48 hours to preserve tissue integrity. Skeletal muscle samples were processed using the PEGASUS protocol, a modified tissue-clearing approach optimized for immunolabeling and three-dimensional imaging. Briefly, tissues were first incubated in a 25% (v/v) solution of Quadrol (Sigma-Aldrich, #122262) at 37 °C on a shaker plate for 2 to 4 days, with daily solution changes. Samples were maintained in the decolorization solution until complete pigment removal was visually confirmed. Following decolorization, tissues were washed in phosphate-buffered saline (PBS) and incubated overnight at room temperature in a commercial blocking buffer supplemented with 2% Triton X-100 to reduce non-specific binding. Primary antibody staining was performed over 3 days at 37 °C under continuous agitation using a blocking buffer containing 0.2% Triton X-100 and the following antibodies at a dilution of 1:1000: goat anti-human VE-cadherin (AF938, R&D Systems) and mouse anti-α-smooth muscle actin monoclonal antibody (A2547, Sigma-Aldrich). After primary incubation, samples were washed in PBS at room temperature for 24 hours with five buffer changes throughout the day. Secondary antibodies, diluted 1:1000 in blocking buffer containing 0.2% Triton X-100, were applied for 3 days at 37 °C on a shaker plate. Donkey anti-goat Alexa Fluor 555 was used to detect VE-cadherin, and donkey anti-mouse Alexa Fluor 647 for α-smooth muscle actin. To preserve fluorophore integrity, all incubations with secondary antibodies were performed in light-protected conditions using aluminium foil. A second round of PBS washes (24 hours, five changes) followed to remove unbound secondary antibodies. Delipidation was conducted through a graded tert-butanol (tB) series at 37 °C under agitation, comprising successive incubations in 30% tB for 4 hours, 50% tB for 6 hours, and 70% tB for 24 hours. Dehydration and refractive index matching were then performed over 48 hours at 37 °C using a tB-PEG solution composed of 70% (v/v) tert-butanol, 27% (v/v) poly(ethylene glycol) methacrylate Mn ~500 (PEG-MMA500, Sigma-Aldrich #447943), and 3% (w/v) Quadrol (Sigma-Aldrich #122262), with one solution change after 24 hours. Samples were then immersed for 2 days at 37 °C in BB-PEG solution containing benzyl benzoate (Sigma-Aldrich #B6630) and PEG-MMA500 for final clearing. All clearing steps were performed with light protection to prevent fluorophore degradation. Cleared samples were stored long-term in BB-PEG at room temperature in the dark until imaging. Labelling with VE-cadherin enabled the identification of endothelial cell borders, referred to here as the intima, while α-SMA immunostaining revealed actin within vascular smooth muscle cells and pericytes, corresponding to the media.

**Hemodynamic model**

The diameter of each generation was set as $d_{i+1}=2^{{-1}/2+\epsilon_{1}}d_{i}$ for the arterial side and $d_{i}=2^{{-1}/2+\epsilon_{2}}d_{i+1}$ for the venular side [17] with ε_1_ and ε_2_ fixed by the diameters of the arterioles, venules and capillaries. The length of each generation was set as $l_{i+1}=2^{{-1}/3-\gamma_{1}}l_{i}$ for the arterial side and $l_{i}=2^{{-1}/3-\gamma_{2}}l_{i+1}$ for the venous side with γ_1_ = -0.06 and γ_2_ = 0.14 [17] and the length of a capillary fixed at 409 µm [18].The flow dynamics were computed following three regimes: Fåhræus-Lindqvist (d > 8 µm), lubrication (d < 7 µm) and mixed (7 µm < d <8 µm). First, for the Fåhræus-Lindqvist regime, the cell-free layer thickness was computed as $r-r'=0.044r+0.74$ with r the radius of the vessel, r’ the radius of the region containing red blood cells, both in µm [2]. Then, all the hemodynamic parameters were computed based on a pseudo-Poiseuille flow with a modified viscosity µ such as $\frac{\mu_{s}}{\mu}=1+\left( \frac{r'}{r} \right)^{4}\left( \frac{\mu_{s}}{\mu_{b}}-1 \right)$ [3] where µ_s_ = 1.68 mPa.s is the viscosity of the serum and µ_b_ = 3.88 mPa.s is the viscosity of the blood [4]. For the lubrication regime, we used Secomb’s formula to compute the viscosity needed for the pseudo-Poiseuille regime : $\frac{\mu}{\mu_{s}}=1+kH_{cap}$ with H_cap_ the hematocrit inside the capillary and k a proportionality factor [5]. The capillary hematocrit H_cap_ was computed with $\frac{H_{cap}}{H}=\sigma^{2}\left[ 1+\frac{\left( 1-\sigma^{2} \right)^{2}}{\sigma^{2}\left( 2-2\sigma^{2}+\sigma^{2}\frac{\mu_{s}}{\mu_{b}} \right)} \right]$ with $\sigma=r'/r$ [3] and H = 0.44 [4] the hematocrit in the large vessels. The k factor was computed as $k=\frac{\pi r^{2}}{V_{RBC}}=\left[ \frac{\Delta pr^{2}}{8\mu\left( u_{0}-2{q_{0}}/r \right)}-h_{RBC} \right]$ [5] with h_RBC_ = 2.14 µm [6] the height of a red blood cell, Δp the pressure drop across the red blood cell, q_0_ the plasma flow relative to the red blood cells, and u_0_ the velocity of the red blood cells relative to the plasma. Δp was calculated as the pressure drop due to a Poiseuille flow with a viscosity µ_s_ and a diameter r across a length of h_RBC_. q0 is calculated via the difference between the hematocrits in the large vessels and the capillaries, and $u_{0}=\frac{q_{0}}{\pi r^{2}}\left( 1-\frac{H_{cap}}{H} \right)^{-1}$ [5]. The hemodynamic parameters were obtained via a Pseudo-Poiseuille flow with the adapted viscosity µ. The intraluminal pressure due to the cells is $\frac{2\mu u_{0}}{r}\left( \frac{2.123ur}{2q_{0}} \right)^{3/2}$ [5] where u is the velocity of the red blood cells. Finally, the transition regime was composed from the two other regimes following a linear transition in the viscosity.

The final diameter of each vessel arises from its deformation due to the internal pressure of the blood. Its value is given by the equilibrium of the elastic force, the internal and the external pressure : $r=\frac{P-P_{ext}r_{0}^{2}}{hE}$ with r the deformed radius, r_0_ the initial radius, P_i_ the pressure inside the vessel, P_ext_ the pressure in the muscle, h the thickness of the wall, and E the equivalent Young modulus of the wall. The equivalent Young modulus is defined as $E=\left( \sum_{i} \left( \frac{h_{i}}{E_{i}} \right)^{-1} \right)\left( \sum_{i} h_{i} \right)$ where E_i_ and h_i_ are the Young modulus and the thickness of each layer. The values of thickness and Young modulus for each layer are given in Table 1. Additionally, we modelled the dilation of the arterioles due to the smooth muscle. We used the data of Dyke et al. [7] who found that the blood flow with vasodilation blocked was equal to 70% of the normal flow. Given the r^4^ dependency of the friction loss, a 30% increase in the flow correspond to a 10% increase in the radius of the vessels. Consequently, the smooth muscle was modelled as a actin layer with a dilation equal to 10% of a passive actin layer [7]. The pericyte density on the capillaries was set at 23.5% [8]. The muscular pressure at rest was set at 15 mm Hg to match the pressure in the venule and the increase of muscular pressure during effort was set at 3.9 mm Hg The flow in the capillary network was fixed to match the red blood cell velocity of 380 µm/s [9].

***Table 1***. Values of thickness and Young modulus for each layer

|  | Height, h (µm) | Young modulus, E (kPa) |
| --- | --- | --- |
| Endothelium | 0.3 [10] | 2.97 [11] |
| Basal membrane | 0.64 - 1.66 (capillaries & venules) [12]  0.3 - 0.055 (arterioles) [13] | 4070 [14] |
| Pericytes | Measured | 6.3 [15] |
| Actin | Measured | 13.7 [16] |

[1] « Wasserman & Whipp’s Principles of Exercise Testing and Interpretation ». Consulté le: 14 mars 2025. [En ligne]. Disponible sur: https://www.wolterskluwer.com/en/solutions/ovid/wasserman--whipps-principles-of-exercise-testing-and-interpretation-5305

[2] B. K. Al‐Khazraji, D. N. Jackson, et D. Goldman, « A Microvascular Wall Shear Rate Function Derived From *In Vivo* Hemodynamic and Geometric Parameters in Continuously Branching Arterioles », *Microcirculation*, vol. 23, n^o^ 4, p. 311‑319, mai 2016, doi: 10.1111/micc.12279.

[3] R. Chebbi, « Dynamics of blood flow: modeling of the Fåhræus–Lindqvist effect », *J. Biol. Phys.*, vol. 41, n^o^ 3, p. 313‑326, juin 2015, doi: 10.1007/s10867-015-9376-1.

[4] « Lowe GDO. Clinical Blood Rheology. 1st ed. Boca Raton : Tayloret Francis Groups; 2019 ».

[5] T. W. Secomb, R. Skalak, N. Özkaya, et J. F. Gross, « Flow of axisymmetric red blood cells in narrow capillaries », *J. Fluid Mech.*, vol. 163, p. 405‑423, févr. 1986, doi: 10.1017/S0022112086002355.

[6] Y. C. Fung, W. C. O. Tsang, et P. Patitucci, « High-resolution data on the geometry of red blood cells », *Biorheology*, vol. 18, n^o^ 3‑6, p. 369‑385, déc. 1981, doi: 10.3233/BIR-1981-183-606.

[7] C. K. Dyke, D. N. Proctor, N. M. Dietz, et M. J. Joyner, « Role of nitric oxide in exercise hyperaemia during prolonged rhythmic handgripping in humans. », *J. Physiol.*, vol. 488, n^o^ 1, p. 259‑265, oct. 1995, doi: 10.1113/jphysiol.1995.sp020964.

[8] R. G. Tilton, C. Kilo, et J. R. Williamson, « and Skeletal Muscle Capillaries », *Microvasc. Res.*, vol. 18, 1979, doi: 10.1016/0026-2862(79)90041-4.

[9] C. R. Honig, M. L. Feldstein, et J. L. Frierson, « Capillary lengths, anastomoses, and estimated capillary transit times in skeletal muscle », *Am. J. Physiol.-Heart Circ. Physiol.*, vol. 233, n^o^ 1, p. H122‑H129, juill. 1977, doi: 10.1152/ajpheart.1977.233.1.H122.

[10] A. R. Pries et W. M. Kuebler, « Normal Endothelium », in *The Vascular Endothelium I*, vol. 176/I, S. Moncada et A. Higgs, Éd., in Handbook of Experimental Pharmacology, vol. 176/I. , Berlin, Heidelberg: Springer Berlin Heidelberg, 2006, p. 1‑40. doi: 10.1007/3-540-32967-6_1.

[11] A. B. Mathur, G. A. Truskey, et W. Monty Reichert, « Atomic Force and Total Internal Reflection Fluorescence Microscopy for the Study of Force Transmission in Endothelial Cells », *Biophys. J.*, vol. 78, n^o^ 4, p. 1725‑1735, avr. 2000, doi: 10.1016/S0006-3495(00)76724-5.

[12] T. Matsubara, M. Ziff, et A. Smith, « Basement membrane thickening of postcapillary venules and capillaries in rheumatoid synovium », *Arthritis Rheum.*, vol. 30, n^o^ 1, p. 18‑30, janv. 1987, doi: 10.1002/art.1780300103.

[13] J. A. G. Rhodin, « The ultrastructure of mammalian arterioles and precapillary sphincters », *J. Ultrastruct. Res.*, vol. 18, n^o^ 1‑2, p. 181‑223, avr. 1967, doi: 10.1016/S0022-5320(67)80239-9.

[14] J. Candiello *et al.*, « Biomechanical properties of native basement membranes », *FEBS J.*, vol. 274, n^o^ 11, p. 2897‑2908, juin 2007, doi: 10.1111/j.1742-4658.2007.05823.x.

[15] S. Lee, A. Zeiger, J. M. Maloney, M. Kotecki, K. J. Van Vliet, et I. M. Herman, « Pericyte actomyosin-mediated contraction at the cell–material interface can modulate the microvascular niche », *J. Phys. Condens. Matter*, vol. 22, n^o^ 19, p. 194115, mai 2010, doi: 10.1088/0953-8984/22/19/194115.

[16] L. Lu, S. J. Oswald, H. Ngu, et F. C.-P. Yin, « Mechanical Properties of Actin Stress Fibers in Living Cells », *Biophys. J.*, vol. 95, n^o^ 12, p. 6060‑6071, déc. 2008, doi: 10.1529/biophysj.108.133462.
